# Revisit linear regression-based deconvolution methods for tumor gene expression data

**DOI:** 10.1186/s13059-017-1256-5

**Published:** 2017-07-05

**Authors:** Bo Li, Jun S. Liu, X. Shirley Liu

**Affiliations:** 1 Department of Biostatistics and Computational Biology, Dana Farber Cancer Institute, Harvard TH Chan School of Public Health, 450 Brookline Ave, Boston, MA 02215 USA; 2 Department of Statistics, Harvard University, 1 Oxford St, Cambridge, MA 02138 USA

We have recently published a statistical deconvolution method to study infiltrating immune cells using tumor RNA-seq data [1]. One of the goals in that work was to understand how proportions of different cell types co-vary across different cancer tissues. To this end, we estimated the abundance of six cell types over 9000 tumor samples across 23 cancer types, and then assessed the correlations of these estimated proportions across the different samples within a cancer type. In particular we compared our method (TIMER) with CIBERSORT [2], a previously published deconvolution approach, for their ability to assess such correlations. To our surprise, we found many non-biological negative correlations between CIBERSORT estimates, and we believed that this artifact was, to a large extent, due to the incorporation of highly similar features in the linear model, or statistical collinearity. Newman et al., the authors of CIBERSORT, have raised concerns that these correlations were due to data normalization, instead of collinearity [3]. While we agree with Newman and coauthors that the forced normalization indeed introduces unwanted negative correlations, we will show in this response that the inclusion of highly similar features contributes as significantly as normalization, if not more, to the observed artificial negative correlations among the estimates obtained by CIBERSORT.

Highly correlated features (covariates) in linear regression models can lead to many technical difficulties, such as high estimation variances, non-robustness, and non-identifiability. Furthermore, it is often misleading to interpret their coefficients at their face value. For example, it is very easy to create examples where when only one of the two highly similar features is included in a regression model, its coefficient is highly significant and positive; whereas when both are included, none of the coefficients is significant or one is positively significant and the other is negative. This issue is a fundamental statistical problem due to lack of information and is unlikely to be solved simply by regularization employed by the CIBERSORT method.

**Fig. 1:**
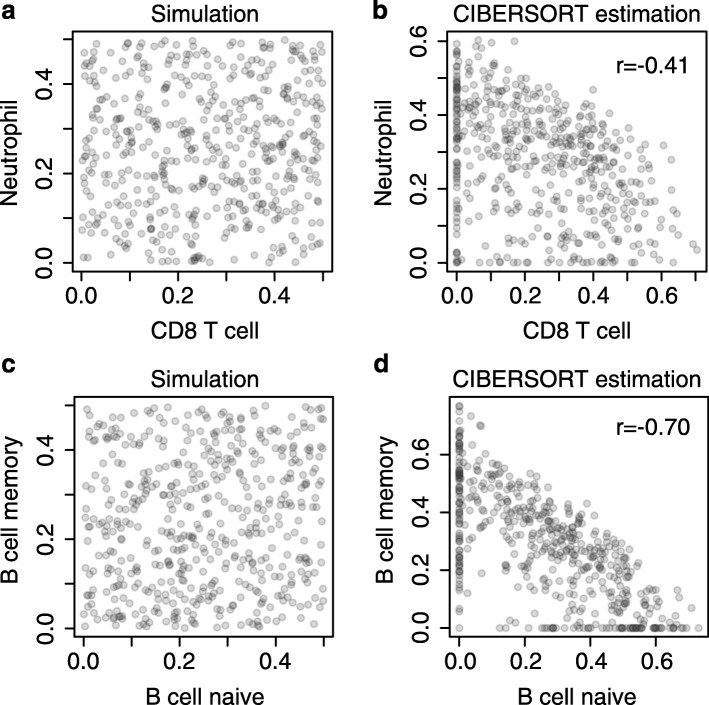
Simulations showing negative associations in CIBERSORT estimates. **a**, **b** CIBERSORT reported a negative correlation between unrelated features, CD8 T cells and neutrophils. The fractions of features were independently sampled from Uniform(0,0.5). **c**, **d** CIBERSORT reported a stronger negative association between closely correlated features, naïve and memory B cells; the sampling procedure used in **a**, **b** was applied. Simulated data were sent to the CIBERSORT online server using LM22 as a reference matrix and default parameters

To evaluate how CIBERSORT estimations are affected by the incorporation of similar features, we conducted two in silico experiments. In the first one, we selected two unrelated cell types, CD8 T cells and neutrophils, from the CIBERSORT feature set, LM22 matrix. The Pearson correlation of the expression levels of the two cell types is 0.009. We generated 500 mixtures by randomly apportioning the population consisting of these two cell types only: **Y** = Y_i_, i = 1,2,…500, where:

$$ {\mathrm{Y}}_{\mathrm{i}}= f{1}_{,} i\kern0.5em  \times \mathrm{X}1 + \kern0.5em  f{2}_{,} i\kern0.5em  \times \mathrm{X}2\kern0.5em  + \varepsilon \mathrm{i} $$

with εi following the normal distribution with mean 0 and the same standard deviation as X1. Coefficients *f1,i* and *f2,i* follow Uniform(0,0.5), and X1 and X2 are respectively CD8 T cell and neutrophil gene expressions from the LM22 matrix. This approach is very similar to the procedure described in the CIBERSORT performance evaluation “Analysis of multicollinearity” [2]. We applied CIBERSORT to estimate the fractions of all the 22 cell types, including CD8 T cells and neutrophils, with the simulated data (Fig. 1a, b). Although *f1* and *f2* were independently simulated, their corresponding CIBERSORT estimates were negatively correlated (*r* = –0.4). Even when the true coefficients (*f1,i*, *f2,i*) were kept as fixed constants across the 500 replications, their estimates continued to show strong negative sampling correlations. This phenomenon is understandable because when one coefficient is estimated higher than its true underlying value, the other one is necessarily estimated lower due to normalization (fixed total sum at 1). This analysis sets the baseline for negative correlation resulting from data normalization in CIBERSORT.

In the second simulation, we replaced CD8 T cells and neutrophils with two highly correlated features: naïve and memory B cells. According to the LM22 matrix, the expression levels are highly correlated (*r* = 0.9). We performed the same simulation and used the online CIBERSORT server to infer *f1* and *f2*. The estimated fractions of the two cell types had even stronger negative correlation (*r* = –0.7; Fig. 1c, d). These results indicate that, in addition to data normalization, the incorporation of highly correlated features will exaggerate the non-biological negative correlations between the estimated coefficients.

In our manuscript [1], we mentioned that some immune subsets may respond to similar cytokines, but this statement was in the context of a previous study which observed such synergy in colorectal cancer [4]. We did not make the argument that, in all cancer types, immune cell levels are correlated, and we agree with Newman et al. [3] that in certain cancers this may not hold true. In their correspondence [3], they made additional analysis of the fractions of each immune subset from flow sorting, which are normalized to the total leukocyte count (Fig. 1a in [3]). Using this approach, Newman et al. [3] observed both positive and negative correlations between different immune subsets. Specifically, naïve B cells and memory B cells were positively correlated (*r* = 0.7), as were active and resting CD4 T cells (*r* = 0.3), which corroborated our speculation that the abundance of closely related cell types may be positively correlated. Since Newman et al. [3] argued that CIBERSORT is compatible with RNA-seq data, we applied it to the The Cancer Genome Atlas (TCGA) RSEM data in several cancer types (Table 1) and focused on the positively correlated B-cell subsets and CD4 T-cell subsets. CIBERSORT found negative correlations for almost all the pairs of related cells in the cancers tested. These negative correlations contradict the experimental results reported in Newman et al. [3]. This result is likely due to the fact that naïve and memory B cells, and similarly active and resting CD4 T cells, have similar expression profiles and are thus difficult to distinguish due to collinearity.

**Table 1:** Associations of CIBERSORT estimates for closely related features

	Naive versus memory B cell (expected r = 0.7)	Activated versus resting CD4 memory T cell (expected r = 0.3)
Glioblastoma	r = −0.34 ⍴ = −0.77	r = −0.05 ⍴ = −0.06
Kidney renal clear cell carcinoma	r = −0.07 ⍴ = −0.29	r = −0.12 ⍴ = −0.13
Lung squamous carcinoma	r = 0.13 ⍴ = −0.38	r = −0.26 ⍴ = −0.19
Lung adenocarcinoma	r = −0.07 ⍴ = −0.37	r = −0.29 ⍴ = −0.26

The expected correlations were obtained from Fig. 1a and are given in the column headings. RNA-seq data of each cancer type were analyzed by the CIBERSORT online server to predict fractions of immune subsets. Pearson’s *r* and Spearman’s ρ were calculated between two pairs of closely related features: (1) naïve and memory B cells, and (2) activated and resting memory CD4 T cells. *Red values* indicate a negative association

The above analyses indicate that incorporating cells with similar features causes CIBERSORT to produce results with non-biological associations.

Estimations from TIMER are proportional to the immune content in the tumor tissue. However, we explicitly emphasized in our manuscript [1] that TIMER estimations “are not comparable between cancer types or different immune cells,” but only comparable across individuals within a cancer type. This is because in TIMER each immune cell type is individually normalized according to the availability of the reference data [5]. The absolute scales of the estimates for different cell types within a tumor are non-biological. In the original manuscript [1], we did not make arguments regarding the estimation of the absolute proportion of a single cell type. Therefore, we are surprised by the claim by Newman et al. [3] that our method reported “disproportionately high levels of typically rare dendritic cells (DCs).” In their correspondence, Newman et al. [3] went ahead and normalized TIMER estimations and made a series of conclusions based on data normalization (Fig. 2d–h in [3]). We believe that these claims resulted from a misinterpretation of our method and data, because the normalization of the total infiltrates to sum to 1 does not apply to TIMER estimates. In addition, we were fully aware that TIMER estimates may correlate with total leukocyte levels (Fig. S3c in [1]). Therefore, in the downstream analyses, we used the partial Spearman correlation conditioning on tumor purity whenever necessary to control for this factor.

**Fig. 2:**
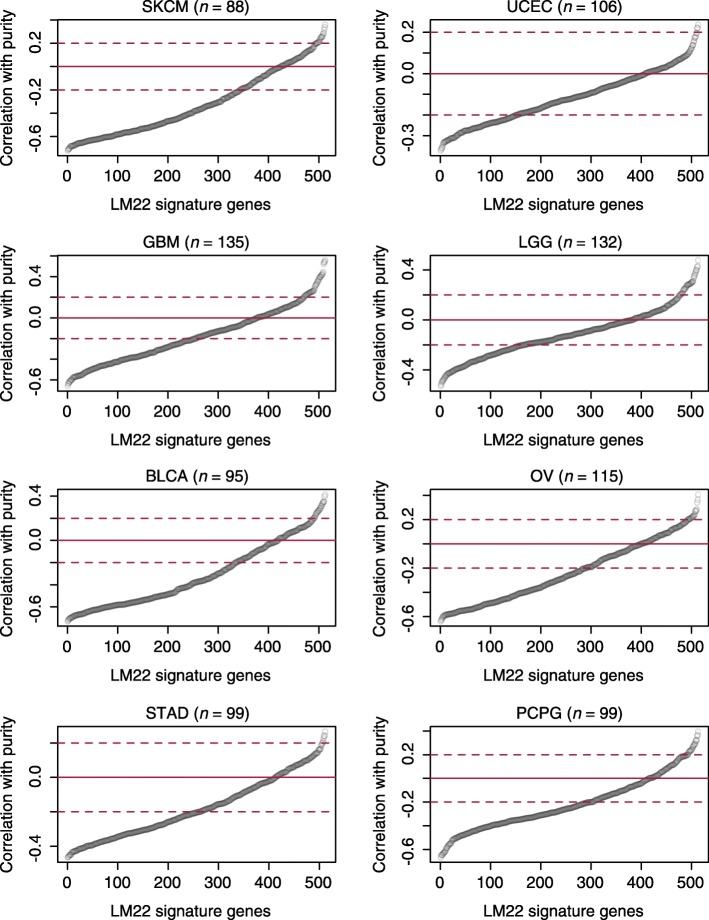
Expression of LM22 signature genes in the malignant cells. For each cancer type, the expression level of each LM22 gene was compared with tumor purity. Positive correlation indicates higher expression in the malignant cells. Genes were ranked by their correlations with purity. As described in the text, a positive correlation between gene expression and tumor purity indicates that the gene is expressed in the malignant cells. Cancer name abbreviations follow The Cancer Genome Atlas nomenclature. *BLCA* bladder cancer, *GBM* glioblastoma, *LGG* lower-grade glioma, *OV* ovarian cancer, *PCPG* pheochromocytoma and paraganglioma, *SKCM* melanoma, *STAD* stomach cancer, *UCEC* uterine endometrial cancer

Let us now revisit the statistical model in CIBERSORT, where the sample mixture is considered as a linear combination of reference features. In this model, gene expression in a tumor tissue sample is a mixture of different immune subsets:

$$ {Y}_g^i={\displaystyle \sum_{j=1}^{22}}{f}_j^i\times {X}_g^i+\varepsilon $$

where *i* indicates the tumor sample, *j* indexes the immune cell type, and *g* stands for an LM22 gene, with constraints ∑_*j* = 1_^22^ *f*_*j*_^*i*^ = 1 and *f*_*j*_^*i*^ ≥ 0, *for* ∀ *i*, *j*. An important hidden assumption of this model is that malignant cells in the tumor tissue do not express a significant amount of any of the LM22 genes, which have been selected a priori based on mRNA expression data profiled from sorted immune cells. However, due to genome instability, it is possible that malignant cells also express immune-related genes.

To examine if this is true, we analyzed the LM22 signature genes using The Cancer Genome Atlas data. We found that, in multiple cancers, a substantial fraction of the 513 LM22 signature genes showed positive correlations with purity (Fig. 2). Such a correlation suggests that samples with higher tumor content express these genes at higher levels, indicating that these genes are also expressed in malignant cells. In their analysis, Newman et al. [2] in silico mixed colon cancer cell line expression data with immune subsets to show that CIBERSORT works for tumor tissues. In our analysis, we found that colon cancer expresses a very small number of LM22 genes (*n* = 56), explaining why CIBERSORT may work well for this cancer type. However, given that up to a quarter of the LM22 genes are not immune-specific in many other cancer types, the model assumption of CIBERSORT is frequently violated. As a consequence, it is likely that the CIBERSORT inferences derived from these genes are confounded by cancer cell expression. This is a possible reason that CIBERSORT [6] failed to identify putative prognostic factors in these cancers, such as T cells in melanoma and ovarian cancer, or macrophages in glioma.

Finally, we would like to re-emphasize that CIBERSORT and TIMER target different aspects of tumor immune infiltrates. CIBERSORT infers the relative fractions of immune subsets in the total leukocyte population, while TIMER predicts the abundance of immune cells in the overall tumor microenvironment. Currently both methods are limited by the assumption that transcriptomes of tumor-infiltrating immune cells do not significantly differ from those collected from peripheral blood of healthy donors. This is a convenient assumption based on practicality but may not hold for many tumors. Future deconvolution methods could continue to improve, with more studies profiling the tumor-infiltrating immune subsets or single-cell tumor transcriptomes to generate high-quality reference data.
